# Relevant principal factors affecting the reproducibility of insect primary culture

**DOI:** 10.1007/s11626-017-0140-7

**Published:** 2017-02-22

**Authors:** Norichika Ogata, Kikuo Iwabuchi

**Affiliations:** 1 Nihon BioData Corporation, 3-2-1 Sakado, Takatsu-ku, Kawasaki, Kanagawa 213-0012 Japan; 2 Laboratory of Applied Entomology, Faculty of Agriculture, Tokyo University of Agriculture and Technology, 3-5-8, Saiwai-cho, Tokyo, Fuchu 183-8501 Japan

**Keywords:** Interindividual variation, Primary explant culture, Coleoptera, Transmission electron microscope, RNA-seq

## Abstract

The primary culture of insect cells often suffers from problems with poor reproducibility in the quality of the final cell preparations. The cellular composition of the explants (cell number and cell types), surgical methods (surgical duration and surgical isolation), and physiological and genetic differences between donors may be critical factors affecting the reproducibility of culture. However, little is known about where biological variation (interindividual differences between donors) ends and technical variation (variance in replication of culture conditions) begins. In this study, we cultured larval fat bodies from the Japanese rhinoceros beetle, *Allomyrina dichotoma*, and evaluated, using linear mixed models, the effect of interindividual variation between donors on the reproducibility of the culture. We also performed transcriptome analysis of the hemocyte-like cells mainly seen in the cultures using RNA sequencing and ultrastructural analyses of hemocytes using a transmission electron microscope, revealing that the cultured cells have many characteristics of insect hemocytes.

**Electronic supplementary material:**

The online version of this article (doi:10.1007/s11626-017-0140-7) contains supplementary material, which is available to authorized users.

## Introduction

Tissue culture has been defined as the maintenance of isolated portions of multicellular organisms in artificial containers outside the individual for considerable periods of time (Murray and Kopech 1953). It was devised in the twentieth century (Harrison et al. 1907; Carrel 1912) to research the behavior of animal cells without the effects of homeostasis and experimental stress present in in vivo experiments (Freshney 2005). Primary explant culture and dissociated cell culture have been used to establish insect cell lines (Lynn 2001). However, the primary culture of insect cells often suffers from problems with poor reproducibility in the quality of the final cell preparations. For example, the freshness of explants markedly affects the quality of cultured cells and explants of poor quality exhibit lower reproducibility (Mothersill et al. 2001; Drobna et al. 2004; Freshney 2005). These problems are obstructive to studying developmental biology and molecular biology in vitro. In this study, we have assessed how interindividual variation affects the development of primary explant cultures by observing 126 fat body explant cultures dissected from six *Allomyrina dichotoma* larvae. That is, we recorded the culture history of each explant culture and analyzed correlations between culture development and time course using linear mixed models. The following two models were constructed: (1) a model containing two random effects, donor individuals and culture replicates, and (2) a model containing only culture replicates; and then the likelihoods of these models were compared.

## Materials and Methods

Japanese rhinoceros beetle *A. dichotoma* last-instar larvae were harvested at Fukutsu-shi, Fukuoka, Japan and reared in leaf mold. All animals used in this study were in diapause. Adult *A. dichotoma* deposit their eggs in suitable leaf mold in August and September, which is the end of the Japanese summer. The eggs absorb water through their surfaces and increase in size. After hatching, the larvae feed on leaf mold and grow to up to 35 g in weight, twice going through ecdysis by November. When winter arrives, *A. dichotoma* enter diapause until spring. The larvae were placed in a 100-mL beaker with 5 mL of 70% ethanol for 30 min and exposed to the vapor. The larvae regurgitated leaf mold from their guts. The surface of the larvae was washed using water and dish detergent (Mama Lemon; Lion Corporation, Tokyo, Japan). The larvae were soaked in 0.1% benzalkonium chloride solution (Nihon Pharmaceutical, Tokyo, Japan) for 30 min. The larvae were then swabbed with 70% ethanol and flame-sterilized. The surface-sterilized larvae were then placed on sterilized filter paper and dissected. The skin on the lateral side of the larval abdomen was cut open along the longitudinal axis using ophthalmic scissors. Fat bodies were removed with tweezers and placed on polystyrene 35-mm dishes (AS ONE, Osaka, Japan). Explants were washed with Shields and Sang M3 insect medium (Sigma-Aldrich, St Louis, MO) containing 10% fetal bovine serum (FBS) using wide-head (*φ* = 3 mm) Pasteur pipettes. Washed explants were transferred into 1 mL of Shields and Sang M3 insect medium (10% FBS) without antibiotics in polystyrene 35-mm dishes (Corning Incorporated, New York, NY). To select a standard culture medium for the primary culture of *A. dichotoma*, we cultured *A. dichotoma* cells using MM medium (Mitsuhashi and Maramorsch 1964), MGM-450 insect medium (Mitsuhashi and Inoue 1988), and Shields and Sang M3 insect medium. In cultures using MM medium, cell migration and proliferation were not observed. In cultures using MGM-450 insect medium, cell migration and proliferation were observed, but the cultures did not become confluent. In cultures using Shields and Sang M3 insect medium, cell migration and proliferation were observed and some cultures became confluent. Cells proliferated in the subcultures and were passaged a maximum of three times. The morphology of the cultured cells was examined using a phase-contrast microscope (Leica DM IRB; Leica Camera AG, Wetzlar, Germany). Cultured cells were categorized by their morphological characteristics: size and roundness (size, larger/smaller than 50 μm, cells of intermediate type were scarcely observed). Additionally, the presence of spindle-shaped cells was noted, because these cells are fast-growing. The morphology of the spindle-shaped cells was similar to that observed in two previously established coleopteran cell lines (Iwabuchi 1999; Hoshino et al*.* 2009). The characteristics of the observed cells were recorded during the time course. In total, four male larvae and two female larvae were used, 126 explants were cultured, and 1223 observations were recorded (Supplementary Material 1). We defined culture developmental phases in order to quantitatively record the progress of these cell cultures over time. All observed cells were categorized by their morphological characteristics: size and roundness (size, larger/smaller than 50 μm, cells of intermediate type were scarcely found throughout the observations; roundness, round-shaped cells having high roundness/slender-shaped cells having low roundness). Cells that were larger than 50 μm with high roundness were classified as Type A. Cells that were larger than 50 μm with low roundness were classified as Type B. Cells that were smaller than 50 μm with low roundness were classified as Type C. Cells that were smaller than 50 μm with high roundness were classified as Type D. Additionally, spindle-shaped cells were noted, because these cells are fast-growing. A culture which contained spindle-shaped cells was defined as Phase 4. Spindle-shaped cells proliferated and if colonies containing more than 30 cells were present (Fig. 2 *D*), the culture was defined as Phase 5. Prior to the appearance of fast-growing spindle-shaped cells, cultures were classified into three phases. In the first phase, type C and type D cells were present. In the second phase, only type D cells were observed. In the third phase, more than three types of cells were present. The progress of the primary explant cultures was quantitatively recorded using these criteria. Data analyses of culture development were performed using the R 2.8.1 (R Core Team 2013) and the lme4 package (Bates et al. 2015). Observation of larval hemocytes using an electron microscope was also undertaken as previously described (Takahashi-Nakaguchi et al. 2010). Total RNA isolation, library preparation, and sequencing for RNA-seq of spindle-shaped cells were performed as previously described (Ogata et al*.* 2012, 2015). Short-read data have been deposited in the DNA Data Bank of Japan’s Short Read Archive under project ID DRA004723. Short reads were assembled using velvet (version 1.1.02; kmer = 63) (Zerbino and Birney 2008) and oases (version 0.1.20) (Schulz et al. 2012) and mapped to the assembled reference data using bowtie (version 0.12.8) (Langmead et al. 2009). Assemble data have been deposited in the DNA Data Bank of Japan’s Transcriptome Shotgun Assembly (IABQ01000000). The homology search and local alignments were determined using BLASTX (BLAST+ version 2.2.25+) (Camacho et al*.* 2009). To examine the expression levels of Notch in the spindle-shaped cell transcriptome, we performed sequence similarity searching using peptide sequences of *Drosophila melanogaster* (Notch isoform A gi|24639454|ref|NP_476859.2|, Notch isoform B gi|386763748|ref|NP_001245510.1|) and *Tribolium castaneum* (NP_001107853.1) as references. Then, we performed sequence similarity searching between nt (non-redundant nucleotide data base of NCBI) and ten contigs from *A. dichotoma* transcriptome data having a similarity with *D. melanogaster* Notch and *T. castaneum* Notch.

## Results

**Figure 1.**
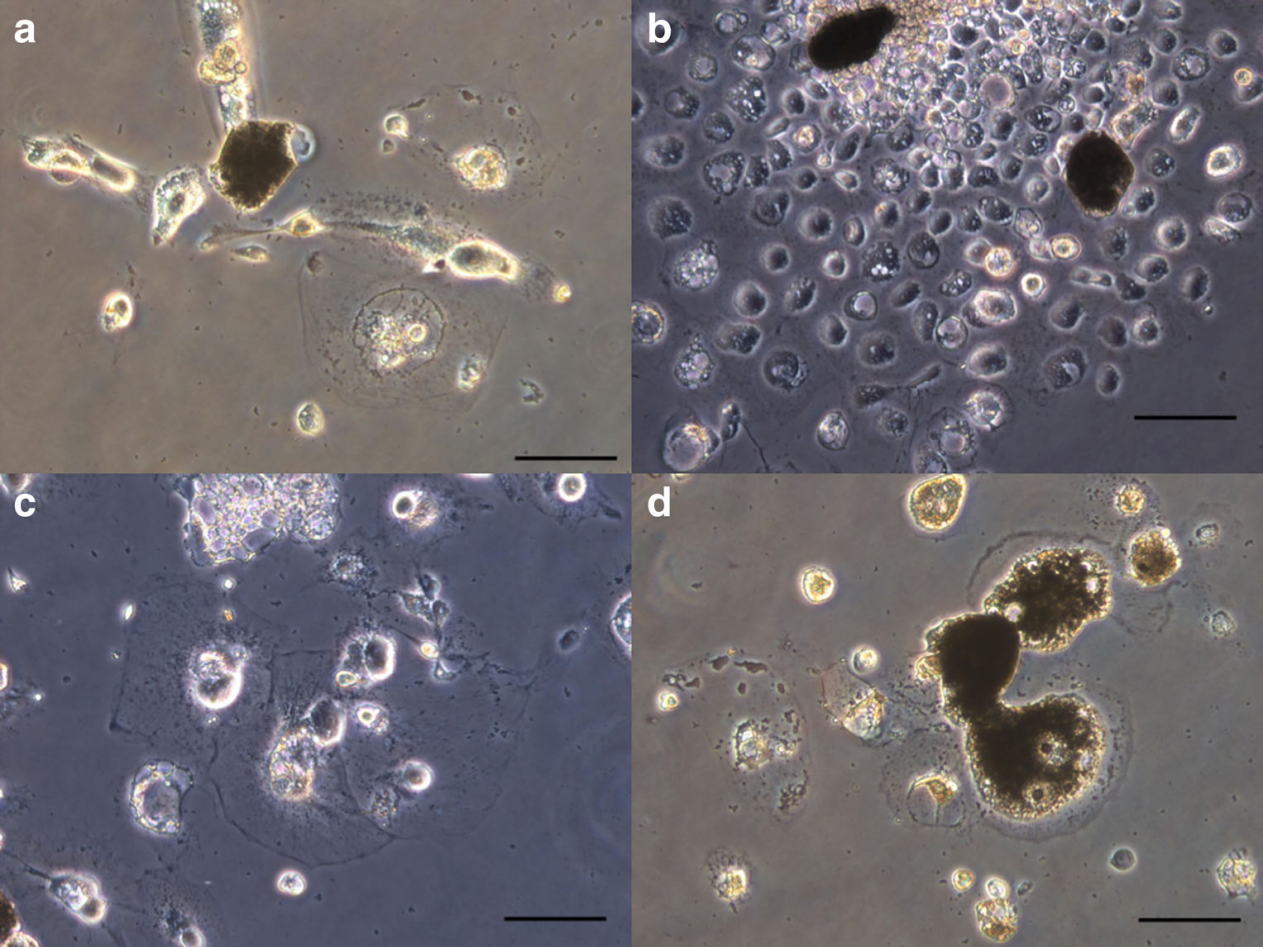
Morphology of plated cells from *A. dichotoma* primary explants. At 48 h after dissection, epithelial cell-like cells were observed. *Scale bar* = 50 μm. All pictures were taken using a phase-contrast microscope.

**Figure 2.**
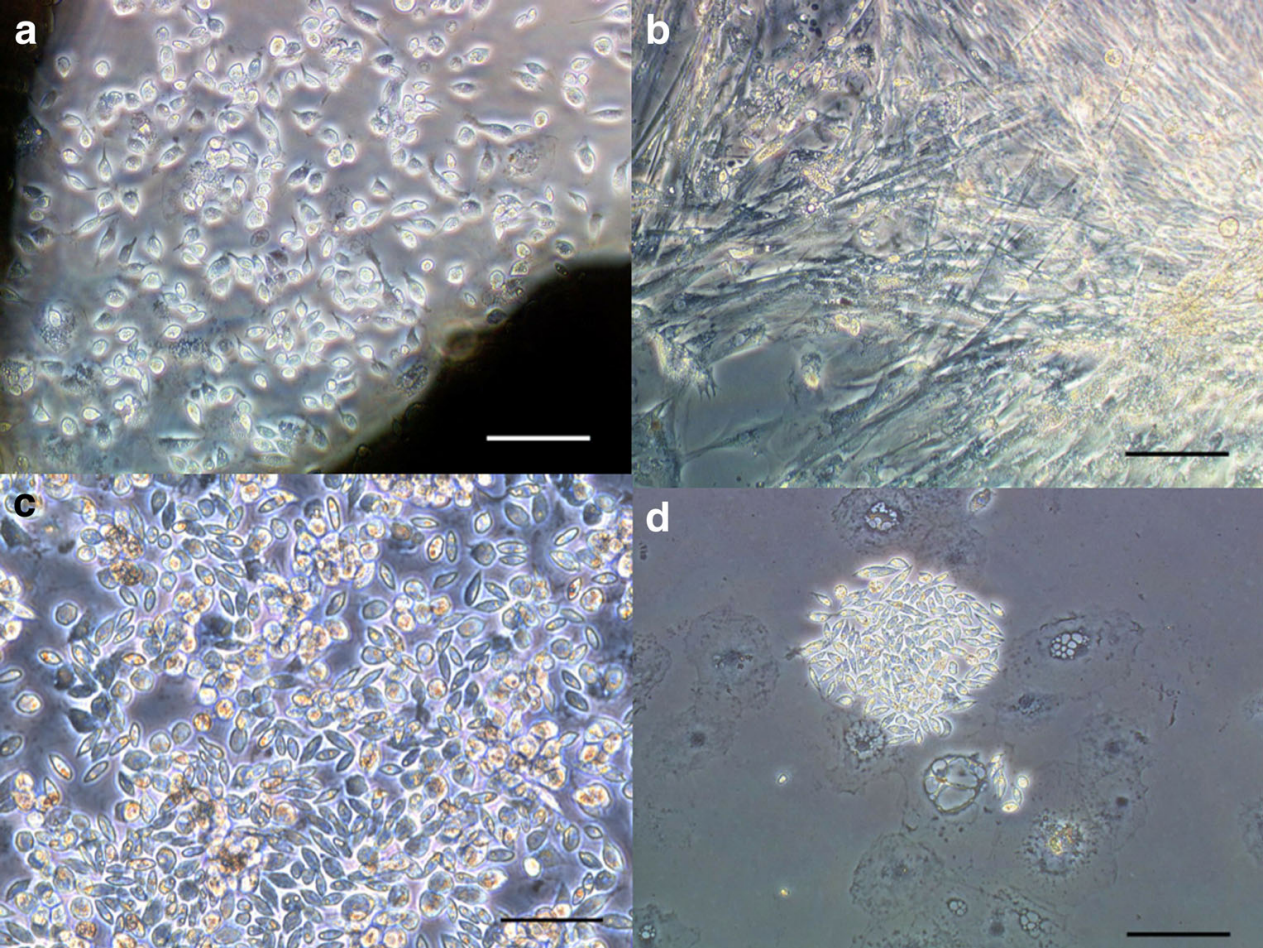
Morphology of cells in *A. dichotoma* primary culture. All pictures were taken using a phase-contrast microscope. (*a*) Hemocyte-like cells. *Scale bar* = 50 μm. (*b*) Fibroblast-like cells. *Scale bar* = 100 μm. (*c*) Spindle-shaped cells. *Scale bar* = 50 μm. (*d*) A colony of spindle-shaped cells. *Scale bar* = 100 μm.

**Figure 3.**
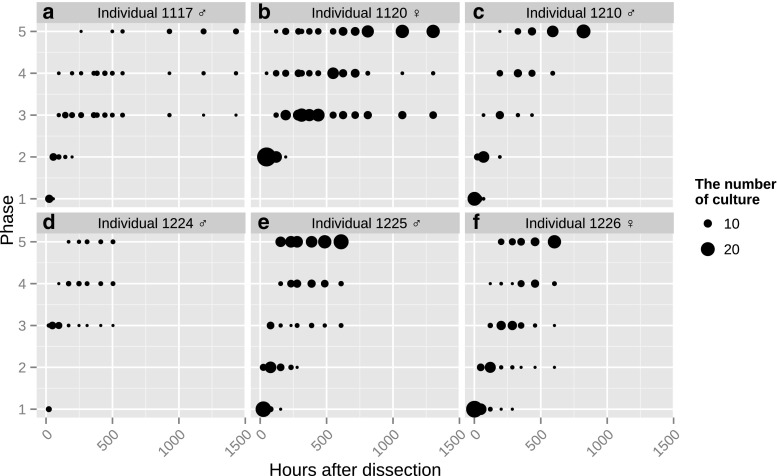
Quantification of primary explant culture development over time. Culture histories of primary cultures isolated from larvae as follows: (*a*) from male larva 1117, (*b*) from female larva 1120, (*c*) from male larva 1210, (*d*) from male larva 1224, (*e*) from male larva 1225, (*f*) from female larva 1226.

We cultured cells derived from six larvae. Immediately after dissection, hemocyte-like cells were observed but disappeared within a few day. Subsequently, epithelial cell-like cells (Fig. 1), hemocyte-like cells (Fig. 2 *a*), and fibroblast-like cells (Fig. 2 *b*) were observed and then, spindle-shaped cells were observed that displayed signs of proliferation (Fig. 2 *c*). Finally, the spindle-shaped cells had increased and some cultures became confluent. Progressions of the primary explant cultures were quantitatively recorded (Fig. 3, Supplementary Material 2).

To test whether a random effect was significant in the development of primary explant cultures, we quantitatively analyzed our observations of primary explant culture histories using a mixed model (Bates et al. 2015). Interindividual and interdish variations were defined as random effects. We constructed first-order, second-order, third-order, fourth-order, fifth-order, and sixth-order equations. The phase of each of the dishes (phase) was explained by the time elapsed since dissection (time), interindividual variation (individual), and interdish variation (dish). The Akaike Information Criterion (AIC) (Akaike 1974) were 3145 (first order eq.), 2978 (second order eq.), 2814 (third order eq.), 2777 (fourth order eq.), 2845 (fifth order eq.), and 2939 (sixth order eq.). The model that has the lowest AIC is optimal; therefore, we selected the fourth-order equation and constructed two models which included different random effects.

A model that included two random effects, the individual and the dish, was constructed in R language:

$$ \begin{array}{l}\mathrm{model}1<\hbox{-} \mathrm{lmer}\\ {}\left(\mathrm{state}\sim \mathrm{I}\left(\mathrm{time}\hat{\mkern6mu} 4\right)+\mathrm{I}\left(\mathrm{time}\hat{\mkern6mu} 3\right)+\mathrm{I}\left(\mathrm{time}\hat{\mkern6mu} 2\right)+\mathrm{time}+\left(\mathrm{time}|\mathrm{individual}/\mathrm{dish}\right),\mathrm{data}=\mathrm{all}\right)\end{array} $$

A second model, which included a random effect and the dish, was constructed in R language:

$$ \begin{array}{l}\mathrm{model}2<\hbox{-} \mathrm{lmer}\\ {}\left(\mathrm{state}\sim \mathrm{I}\left(\mathrm{time}\hat{\mkern6mu} 4\right)+\mathrm{I}\left(\mathrm{time}\hat{\mkern6mu} 3\right)+\mathrm{I}\left(\mathrm{time}\hat{\mkern6mu} 2\right)+\mathrm{time}+\left(\mathrm{time}|\mathrm{dish}\right),\mathrm{data}=\mathrm{all}\right)\end{array} $$

The AIC of model 1 and model 2 was 2729 and 2873, respectively. Likelihood ratio testing revealed that model 1 explained the culture histories better than model 2 (*p* < 0.001). The estimated value of random effects was 0.53 (individual, time-independent), 0.61 (dish, time-independent), 8.7 × 10^−3^ (individual, time-dependent), and 3.8 × 10^−3^ (dish, time-dependent). These results show that interindividual variation affects the initial state of development of primary explant cultures, which implies a poor reproducibility of primary explant culture. Cultured explants of *A. dichotoma* larvae dissected in March and April demonstrated wider interindividual variation (data not shown).

**Figure 4.**
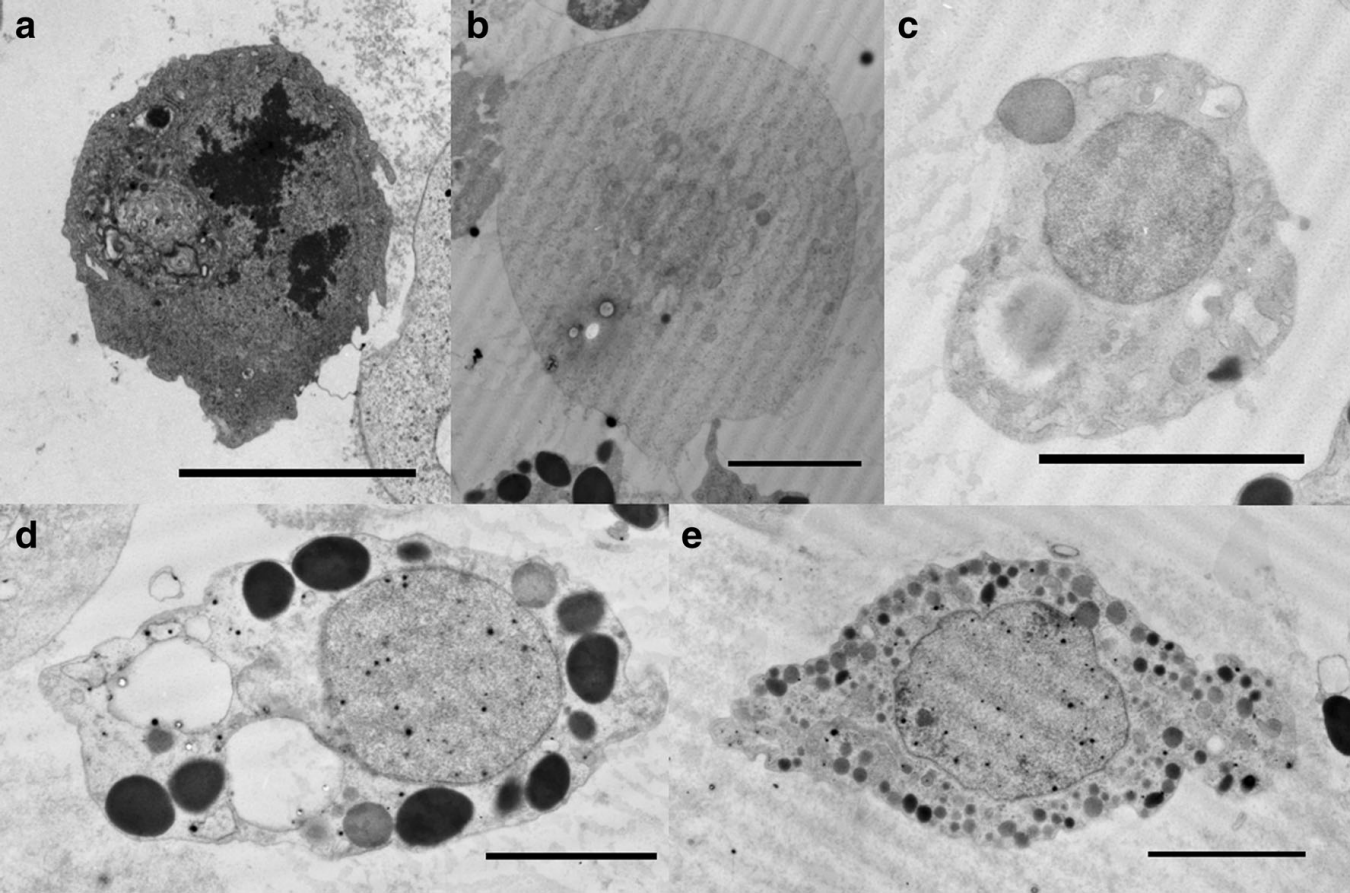
Ultrastructures of *A. dichotoma* larval hemocytes. Electron microscope analyses of larval hemocytes were performed using a transmission electron microscope. *Scale bar* = 8 μm. (*a*) Proleucocyte. (*b*) Oenocytoids. (*c*) Spherule cell. (*d*) Granule cell. (*e*) Plasma cell.

Immediately after dissection, hemocyte-like cells (granulocyte-like and plasmatocyte-like cells) were observed. Other types of hemocytes that were not adherent might have been removed during explant washing. Electron microscope analyses of larval hemocytes from the Japanese rhinoceros beetle revealed the presence of proleucocytes, oenocytoids, spherule cells, granule cells, and plasma cells (Fig. 4).

Our previous study revealed that cultured *Bombyx mori* fat bodies dedifferentiate 80 h after dissection (Ogata et al. 2012). Our statistical analysis showed that interindividual variation affects the initial state of development of primary explant cultures. The interindividual variation detected in this study could be explained by the interindividual dedifferentiation ability within cells derived from individual animals which would have variations in cell physiology when harvested.

## Discussion

In this study, cell migration from explants and cell proliferation were observed using *A. dichotoma* larval fat body cells cultured with Shields and Sang M3 insect medium containing 10% FBS. Despite the fact that Coleoptera is the largest order in the animal kingdom, cell lines are vastly underrepresented. Cell culture media which have been successfully used include MGM-450 insect medium, DCCM media, Schneider’s B media, Shields and Sang M3 insect medium, and EX-CELL 400 (Barcenas et al*.* 1989; Mitsuhashi 1989; Lynn 1995; Fernon et al. 1996; Iwabuchi 1999; Charpentier et al. 2002; Mitsuhashi 2003; Hoshino et al. 2009).

The top ten expressed genes in spindle-shaped cells were similar to that of the hemocytin, alpha-l1 nicotinic acetyl choline receptor, tubulin alpha-1 chain, apolipophorins, and polyadenylate-binding protein 4-like isoform ×1 (Supplementary Material 3). There was no common gene between these ten genes and previously reported differential expressed genes in response to wounding and/or immune challenge in an insect (Johnston and Rolff 2013). Hemocytin is an adhesive protein and roles in hemostasis or encapsulation of foreign substances for self-defense have been suggested (Kotani et al. 1995). This result supports the idea that the spindle-shaped cells proliferating in our primary explant cultures of *A. dichotoma* were hemocyte-like cells. The cells migrating from the explants sometimes attached to the surface of the culture dishes and showed rhythmical peristalsis (Supplementary Material 4). During embryonic development in amniotes, mesodermal cells generate smooth muscle cells and hemocytes (Shin et al. 2009). The migrating cells seen in this study showed a similarity to mesodermal cells. It is known that levels of Notch activity influence the segregation of smooth muscle cells and blood/endothelial cells (Shin et al. 2009) and Notch-activated mesodermal cells are biased to become smooth muscle cells. Levels of Notch activity in the spindle-shaped cells proliferating in our primary explant culture are therefore likely to be low. To investigate this hypothesis, we examined the expression levels of Notch in the spindle-shaped cell transcriptome. As a result, we found that the genes which have the highest sequence similarity with those ten contigs were not Notch (they included LAMB1, drpr, and fibrillin). Those ten contigs with a partial similarity with Notch were not homologous genes of Notch. These results suggest that expression levels of Notch in the spindle-shaped cells were below the detectable limit.

## Conclusions

Interindividual variation suggests high cell variability, and therefore, poor reproducibility of primary explant culture. Variability of cell types and cell responses makes obtaining consistent results in primary cell culture efforts difficult. Proleucocytes, granule cells, plasma cells, oenocytoids, and spherule cells were observed in *A*. *dichotoma* larval hemolymphs. The top genes expressed in the selected cells and proliferated in *A. dichotoma* larval fat body primary culture were supportive of reports for hemocytes.

## Electronic supplementary material

ESM 1 Observations in this study. In total, four male larvae and two female larvae were used, 126 tissues were cultured, and 1223 observations were recorded. (TXT 427 bytes)

ESM 2 Progression of the primary explant cultures. Culture histories of primary cultures isolated from larvae. (TXT 28 kb)

ESM 3 Most expressed ten transcripts in spindle-shaped cells. List of most expressed ten transcripts in spindle-shaped cells with nr BLASTX hits, Accession numbers, and E-values. (TXT 1 kb)

ESM 4 Rhythmical peristalsis of cells which have migrated from the explant. Cells migrated from an explant and attached to the surface of the culture dish. They demonstrated rhythmical peristalsis. The movie was made using a phase-contrast microscope. (MPG 108170 kb)
